# Consequences of COVID-19 Lockdown on the Misuse and Marketing of Addictive Substances and New Psychoactive Substances

**DOI:** 10.3389/fpsyt.2020.584462

**Published:** 2020-10-23

**Authors:** Annagiulia Di Trana, Jeremy Carlier, Paolo Berretta, Simona Zaami, Giovanna Ricci

**Affiliations:** ^1^Unit of Forensic Toxicology, Section of Legal Medicine, Department of Excellence of Biomedical Sciences and Public Health, Polytechnic University of Marche, Ancona, Italy; ^2^Unit of Forensic Toxicology, Section of Legal Medicine, Department of Anatomical, Histological, Forensic, and Orthopedic Sciences, Sapienza University of Rome, Rome, Italy; ^3^National Centre on Addiction and Doping, National Institute of Health, Rome, Italy; ^4^Medico Legal Section, School of Law, University of Camerino, Macerata, Italy

**Keywords:** New Psychoactive Substances, social isolation, COVID-19, SARS-CoV-2, early warning advisory systems, socio-economic crisis

## Introduction

Since the first outbreak of SARS-CoV-2 (severe acute respiratory syndrome-coronavirus-2) in China in December 2019, the infection has rapidly spread all over the world. This new virus has caused many cases of Coronavirus disease 2019 (COVID-19), a potentially fatal respiratory syndrome (1). Due to its global diffusion, the World Health Organization rapidly issued an international warning and declared a worldwide pandemic in March 2020. Currently, most countries are experiencing COVID-19 outbreaks with new infections and fatalities every day and all over the world (2). Due to the mode of transmission of the virus via droplets or direct contact, governments were compelled to adopt restrictive strategies to contain the pandemic and preserve the public health (2, 3). These interventions include limited international mobility, temporary closure of non-essential businesses and more stringent measures like social distancing or complete isolation for prolonged periods. Therefore, this unprecedented crisis has seriously impacted the global economy and people's daily life.

The market of addictive substances has been impacted from the production to the distribution, modifying consumption patterns. An increased consumption of cannabis products and benzodiazepines was reported due to the general feeling of stress caused by the pandemic and associated restrictions, while a decrease in the demand of stimulants was observed due to the inaccessibility of usual recreational settings (4). Moreover, drug misuse may have shifted toward alternative substances and home-made New Psychoactive Substances (NPS) (5–7), which consist of molecules, like pharmaceutical drug analogs, research chemicals and prescription drugs eliciting the psychoactive effects of common illicit addictive drugs or prescription pharmaceuticals (8, 9). The current situation is complex due to the heterogeneity of policies applied in diverse countries and the drugs involved. In this concern, the drug market is constantly monitored by international agencies, such as the United Nation Office on Drug and Crime (UNODC), the European Monitoring Centre on Drugs and Drug Addiction (EMCDDA) and Europol, which collaborate to form a crucial network to prevent the emergence of new dangerous trends.

In this article, the authors critically discuss the most recent data on the impact of COVID-19 on the illicit trafficking of substances and the possible developments of NPS trends in the near future. The authors also draw the attention on the essential role of international networking against drug misuse, especially in times of global crisis.

## Impact of International Restrictions on Drug Production, Trade and Market

The anti-COVID-19 restrictive measures have impacted the drug production in a different manner depending on the substance.

The cultivation of natural drugs is usually conducted in different regions of the world during different periods of the year, depending on the climate. Accounting for 84% of the world production, Afghanistan is the main producer of opium in the Golden Crescent, where poppy is usually harvested between March and June (10). However, the travel restrictions adopted this year have impeded the recruitment of poppy lancers from other regions, and the workforce shortage slowed down harvesting, leading to a partial loss of the production (5). In other countries such as Myanmar, opium harvesting was completed but a decrease in the number of customers was reported (10). Furthermore, the closure of Myanmar borders may have affected the import of acetic anhydride, impacting the production of heroin. Meanwhile, other factors have affected the cultivation of cocaine, which is mainly conducted in Colombia (70% of the global cultivation), Peru (20%), and Bolivia (10%) (10). Since coca leaves can be harvested throughout the year, the anti-COVID-19 measures have not impacted harvesting in those countries. However, the law enforcement pressure hike during the COVID-19 pandemic and the shortage of essential chemical precursors, such as permanganate salts, and gasoline resulted in the reduction of the production of cocaine, especially in Colombia (5). To date, the production of cocaine seems to be less affected in Peru, but the price reductions suggest that large quantities of drugs were stockpiled (5). Since cannabis products are often locally produced and distributed through short supply chains, the production and distribution of cannabis has not suffered due to the global restrictions (5, 6, 11).

A different pattern was observed for synthetic drugs, whose production is less related to the geographical location, and probably because clandestine manufacturing laboratories need less workforce. Amphetamine-type stimulants [i.e., methamphetamine, amphetamine and methylenedioxymethamphetamine (MDMA)] are the most commonly used synthetic drugs, and the bulk production is concentrated in few countries only. According to recent data, laboratories manufacturing synthetic drugs are mainly located in North America (84%), followed by Europe and Asia (10). It is noteworthy that the production of synthetic drugs strictly depends on the availability of chemicals, usually imported from China. Therefore, international travel restrictions and the disruption of raw material production may pose a problem. In fact, a decrease in the availability of synthetic drugs was reported in various countries (e.g., amphetamine in Czech Republic, Lebanon and Syria, and fentanyl and methamphetamine in Mexico) (5, 6).

Drug availability also depends on trafficking routes. The complete interruption of air traffic especially affected the export of synthetic drugs from South East Asia and Oceania. Cocaine trade was less impacted by air travel disruption, due to the use of the maritime route (5, 6). Furthermore, cocaine export from South America is usually conducted by yacht and other modified boats. Air trafficking may have been replaced by postal distribution, wherever it is possible. Maritime trafficking may also have been preferred to bypass COVID-19-related land controls. In this concern, South-Eastern Asian heroin trafficking has shifted from land to maritime transportation across the Indian Ocean. The highest impact of the global trade disruption is expected for the substances that are usually transported along with licit goods, such as heroin and synthetic drugs (5, 10). In recent years, specialized websites have appeared on the darknet as an alternative way to obtain illegal products. Even though several markets have closed since 2018, the darknet still plays a key role in the worldwide diffusion of NPS (6, 12, 13). Although the drugs found on the darknet represent 0.2% of the retail sales in western countries, a sharp increase of the darknet drug trade was reported in Europe during the first 3 months of 2020 (10, 11). According to a preliminary study, cannabis-related products are the most sold merchandises through specialized darknet websites in Europe (6, 11).

As a result, the drug market has been affected differently at retail and bulk levels. A shortage of several types of drugs and a reduction of their purity was reported in many countries. For example, heroin completely disappeared from street markets in Czech Republic. Conversely, bulk distribution appeared more heterogeneous, with a decrease in seizures in several countries including Italy, Niger, and Central Asia, but an increase in other countries such as Iran and Morocco (5, 6, 10). However, this discrepancy may depend on the local anti-Covid-19 restrictions and the difference in commitment to enforce these restrictions.

## Discussion

During this year, the SARS-CoV-2 pandemic has posed various challenges to the population. Fear, stress, and anxiety have affected people all over the world, exacerbating latent psychiatric and psychological disorders (14). Furthermore, the general feeling of uncertainty is fueled by the probable economic crisis that will result from the disruption of non-essential businesses in most countries (15). Fragile categories such as people with drug use disorder suffer from the life-style changes, posing additional public health concerns (16).

Besides, the anti-COVID-19 restrictive measures modified the drug offer and altered substance misuse patterns. Drug-related phenomena like the drugs-and driving and drug parties are expected to decrease (17, 18). Due to high addiction liability, we suppose that the global shortage of heroin may have forced regular users to take other substances with similar effects, such as fentanyl analogs. Moreover, the low quantity of heroin available may have been adulterated with other psychotropic molecules to obtain more potent mixtures at cheaper costs (7, 19). In our opinion, the production of new NPS and NPS use are also expected to increase due to several factors. Firstly, the disruption of the marketing of specific chemical precursors may have forced drug manufacturers to find alternatives, as observed with “Sisa,” a drug that emerged onto the Greek market during the economic crisis of 2010 (20). Secondly, the decrease in the importation of chemical precursors may have favored the domestic manufacture of domestic precursors, as observed with mephedrone in Russia (10). Another important factor to consider is the increase of law enforcement controls that are not suited for the detection of new uncontrolled molecules. Recently, the intentional misuse of prescription drugs to induce psychotropic effects has spread among people with substance use disorder. The most common misused molecules include gabapentinoids, fentanyl analogs, approved antipsychotics, antidepressants and performance-enhancing drugs (21). For this reason, the diversion of prescription drugs like benzodiazepines, opioids and cognitive enhancers is expected to increase due to higher availability (22–24).

Local governments should implement effective measure to prevent those trends that could worsen the state of public health systems. As suggested by Zaami et al., the continuation of drug treatment services along with the implementation of psychiatric and psychological assistance to people with drug use disorder should be ensured to reduce harm (16). To date, a constructive international network is continuously working to monitor the drug market (15, 16).

Since its establishment in 2007, the UNODC combats drug misuse and illicit trafficking through research, guidance and support to governments (25). Common international treaties such as The Single Convention on Narcotic Drugs of 1961 and the Convention on Psychotropic Substances of 1971 were issued by the UNODC and are regularly incremented. Following the emergence of the alarming phenomenon of NPS, the UNODC Early Warning Advisory (EWA) was launched in 2013. The first aim of EWA is to monitor, analyze and report the trends of psychotropic substances that are not included in the above-mentioned international conventions (26). The base of the successful work of the UNODC EWA is the tight collaboration with national and regional agencies and governmental entities (26).

The EMCDDA is a partner of UNODC EWA and coordinates the European network against NPS. In 1997, the Early Warning System (EWS) was implemented under Joint Action 97/396/JHA as a response to the growing NPS concern (27). To EWS is based on a multidisciplinary network comprising several agencies, such as EMCDDA, Europol, the European Medicines Agency (EMA) and 30 national early warning systems. Each national agency operates according to the most recent Regulation (EU) 2017/2101 that establishes a common risk-assessment procedure and a shared three-step approach to respond to NPS (27). In this framework, EMCDDA collects and analyzes national data on NPS emergences, seizures and poisonings to compile a biannual report. Data are also shared with UNODC for a more comprehensive analysis. The European national systems are independent and each state is responsible for its functioning. In Italy, the National Early Warning System (SNAP) on NPS is managed by the National Centre on Addiction and Doping of National Institute of Health (ISS). In this concern, an online platform was developed to allow collaborating centers to spread across the territory to promptly transmit NPS-related information. In addition, EMCDDA data on NPS are reported to SNAP to ensure information sharing between European countries (28).

This capillary network has proved necessary to constantly monitor the new trends of the NPS erratic market. However, many of the current tools for monitoring drug issues at national and international levels are old and may be not effective to capture the complexity of the new drug market. In this concern, the international community should implement more powerful instruments to preserve public health, especially in critical situations such as the COVID-19 pandemic.

## Author Contributions

All authors equally contributed to the conceptualization, preparation, and revision of the paper.

## Conflict of Interest

The authors declare that the research was conducted in the absence of any commercial or financial relationships that could be construed as a potential conflict of interest.
